# Association between polychlorinated biphenyl (PCB) and dioxin with metabolic syndrome (METS): a systematic review and meta-analysis

**DOI:** 10.1038/s41598-024-68369-9

**Published:** 2024-08-02

**Authors:** Mohd Danial Mohd Efendy Goon, Sarah Zulkifli, Siti Suhana Abdullah Soheimi, Sharaniza Ab. Rahim, Normala Abd Latip, Norbaya Hashim, Nirmala Devi Kerisnan, Nasehir Khan E. M. Yahaya, Alias Mohamed, Siti Hamimah Sheikh Abdul Kadir

**Affiliations:** 1 Institute of Pathology, Laboratory and Forensic Medicine (I-PPerFoRM), Universiti Teknologi MARA (UiTM), Sungai Buloh Campus, 47000 Sungai Buloh, Selangor Malaysia; 2 Institute of Molecular Medicine Biotechnology (IMMB), Faculty of Medicine, Universiti Teknologi MARA (UiTM), Sungai Buloh Campus, 47000 Sungai Buloh, Selangor Malaysia; 3 Department of Biochemistry and Molecular Medicine, Faculty of Medicine, Universiti Teknologi MARA (UiTM), Sungai Buloh Campus, 47000 Sungai Buloh, Selangor Malaysia; 4 Integrative Pharmacogenomics Institute (iPROMISE), Universiti Teknologi MARA (UiTM), Puncak Alam Campus, Puncak Alam, 40450 Shah Alam, Selangor Malaysia; 5 National Water Research Institute of Malaysia (NAHRIM), Lot 5377, Jalan Putra Permai, 43300 Seri Kembangan, Selangor Malaysia; 6 Sewerage Service Department (JPP), Block B, Level 2 and 3, Atmosphere PjH No 2, Jalan Tun Abdul Razak, Precinct 2, 62100 Federal Territory of Putrajaya, Malaysia

**Keywords:** Polychlorinated biphenyl, Dioxin, Obesity, Hypercholesterolemia, Hypertension, Diabetes mellitus, Metabolic syndrome, Biochemistry, Environmental sciences, Medical research

## Abstract

Polychlorinated biphenyls (PCBs) and dioxin are persistent endocrine disrupting chemicals (EDCs) and have been associated with an increased risk of metabolic syndrome (MetS). The aim of this systematic review and meta-analysis was to assess the associations of PCBs and dioxin with MetS and its risk factors, including obesity, hypertriglyceridaemia (HTG), hypertension (HTN) and diabetes mellitus (DM). We searched three electronic databases for epidemiological studies concerning PCBs and dioxin with MetS published up to the end of 2023. Meta-analysis was performed for MetS itself and each of the MetS risks based on a random-effects meta-analysis model, and odds ratios (ORs) with 95% confidence intervals (CIs) were obtained. Publication bias was assessed based on Egger’s test. Eleven studies were included from three databases up to 2023. There were 40,528 participants aged 18–89, where 18–100% of them were males, included in our meta-analysis. The meta-analysis results showed a strong association between PCB exposure and DM (OR = 3.593, 95% CI 2.566, 5.031), while most of the risk factors for MetS, including obesity (OR = 1.875, 95% CI 0.883, 3.979), HTN (OR = 1.335, 95% CI 0.902, 1.976) and HTG (OR = 1.611, 95% CI 0.981, 2.643), were weakly associated with PCB. Furthermore, both PCBs (OR = 1.162, 95% CI 0.994, 1.357) and dioxin (OR = 2.742, 95% CI 1.936, 3.883) were found to be weakly and strongly associated with MetS, respectively. Meta-regression analysis showed that DM in the Asian population is associated with PCB exposure, while HTG in the Northern American population is associated with PCB exposure. Our meta-analysis has demonstrated a strong relationship between DM and PCBs, while the relationship between PCBs with MetS and other risk factors is less pronounced. Additionally, MetS is weakly associated with dioxin exposure. To improve primary care outcomes, healthcare providers should consider incorporating the assessment of patients' risk of exposure to PCBs and dioxins into their evaluation procedures for more targeted medical interventions.

## Introduction

Metabolic syndrome (MetS) can generally be defined as a combination of five risk factors in a patient. The five risks are insulin resistance with or without glucose intolerance, abdominal obesity, increased blood pressure (hypertension [HTN]), atherogenic dyslipidemia and prothrombotic and proinflammatory states^1^. Few organizations have established respective criteria in stratifying the risk factors to establish the diagnosis of MetS in a patient. The criteria outlined by the National Cholesterol Education Program’s Adult Treatment Panel III (NCEP:ATP III) to diagnose MetS in a patient should fulfil three or more criteria^2^, such as being obese with a waist circumference greater than 102 cm for males and greater than 88 cm for females, dyslipidemia with triglyceride levels exceeding 1.7 mmol/L, high-density lipoprotein (HDL) levels less than 1.0 mmol/L for males and less than 1.3 mmol/L for females, HTN with readings exceeding 135/85 mmHg and a diagnosis of glucose intolerance with fasting plasma glucose levels greater than 6.1 mmol/L^3^. The consequence of developing MetS in a patient is the increased risk of developing associated diseases such as narrowing of blood vessels known as atherosclerosis^3^ and cancer^4^.

Many in vitro studies have demonstrated the effects of endocrine-disrupting chemical (EDC) exposure in increasing the risk factors for MetS, such as obesity^5^, diabetes mellitus [DM]^6^, and heart-related diseases^7^. For example, congeners of polychlorinated biphenyl (PCB) have been shown to promote obesity by increasing adipocyte resistance towards apoptosis, where adipocytes provide space for PCB accumulation and lead to altered TNF-α expression, which may eventually inhibit apoptosis^8,9^. EDCs are man-made chemicals that pollute rivers and are found in potable water^10,11^. Hence, many studies have suggested that humans are mainly exposed to EDCs through daily drinking water^12^. Furthermore, water containing EDCs is ultimately channelled to industrial and agricultural areas to produce consumables and agricultural products^13,14^.

EDCs are found in plastic containers and food packaging^15^, canned foods^16–18^, and children toys^19^. These studies suggest that exposure to EDCs involves every stage of life, but the dearth of comprehensive research on the impact of PCBs and dioxins on vulnerable populations, including children, is evident^20^. Further, the dose–response relationships between PCB and dioxin exposure and the risk of MetS, are yet to be explored^21^. This comprises the discernment of specific thresholds at which unfavorable outcomes materialize. Moreover, the health implications of protracted, low-level exposure to PCBs and dioxins have not been extensively recorded^22^. Importantly, the negative effects of EDC exposure on humans and the risk of developing MetS are still not well understood, despite many studies have been performed on in vitro and in vivo models. While some meta-analyses have assessed the relationship between the incidence of DM and PCB and its congeners dioxin^23,24^, data on the relationship between these contaminants and the burden of MetS itself are lacking primarily among human population studies that were exposed to these persistent organic pollutants (POPs). Hence, they were reviewed in the present systematic review and meta-analysis, considering the substantial global prevalence of MetS, which ranges from 12.5 to 31.4% depending on the definition used^25^.

## Materials and methods

**Figure 1:**
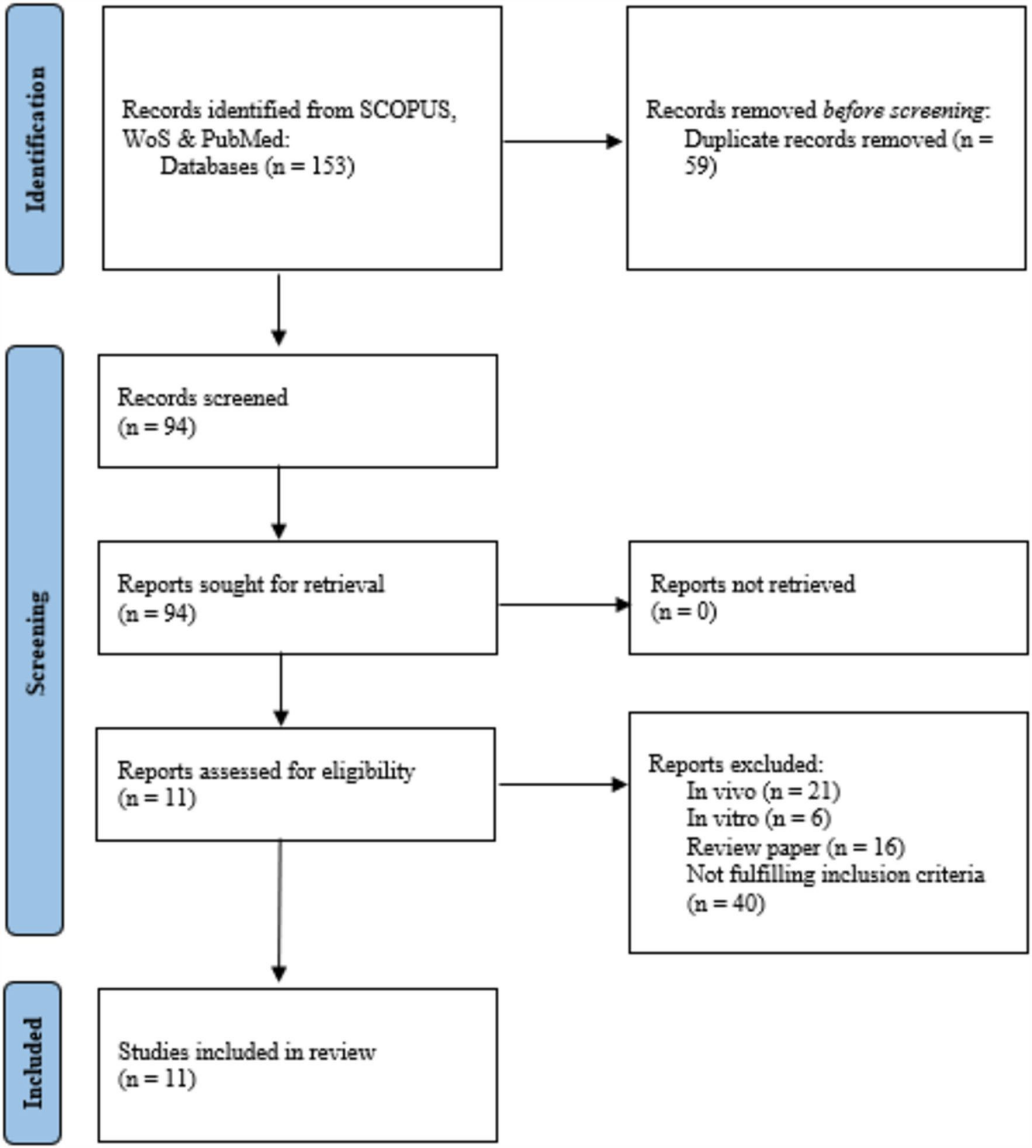
Flow chart of the search strategy for articles using the PRISMA guidelines.

A systematic review and meta-analysis (Fig. 1) was carried out according to the guidelines outlined by the Meta-analysis of Observational Studies in Epidemiology^26^ and the criteria outlined by the Preferred Reporting Items for Systematic Review and Meta-analyses (PRISMA; Supplementary Information 1)^27^. This work has been registered on INPLASY (International Platform of Registered Systematic and Meta-analysis Protocols) and the registration number is INPLASY202450047. Nevertheless, this review was conducted in a rigorous and transparent manner, adhering to established ethical and methodological standards for systematic reviews and meta-analyses, as stated above.

### Search strategy

A literature search was performed by two researchers independently using three online databases, SCOPUS, Web of Science (WoS) and PubMed, on articles related to PCB and its congeners dioxin and furans with the outcome of MetS. The year of the articles was limited between 2017 and 2023. We focused only on the more recent evidence because it has been suggested that the current information on the relationship between EDC exposure and metabolic abnormalities is heterogeneous and fragmented^28^. The articles were identified using the string method in each database, which included the keywords “persistent organic pollutant” OR dioxin OR “dioxin-like polychlorinated biphenyl” OR “dioxin-like compound” OR “polychlorinated dibenzodioxin” OR “polychlorinated dibenzo-p-dioxin” OR “polychlorodibenzo-4-dioxin” OR PCDD OR TCDD OR tetrachlorodibenzodioxin OR tetrachlorodibenzodioxin OR chlorodibenzofuran OR “polychlorinated dibenzofuran” OR “chlorinated dibenzofuran” OR “polychlorinated biphenyl” OR PCB OR “polychlorobiphenyl compound” AND “metabolic syndrome” (Supplementary Information 2). Independent research for each risk factor for MetS in the databases was not performed because those risks were analysed as separate outcomes instead, where articles that fell under the ‘metabolic syndrome’ keyword only will be considered for the analyses. The searches were performed within topics (article title, abstract and keywords), and the articles included were limited only to English articles and human studies. The articles identified using the keywords were further assessed manually by two researchers to further identify the articles to be included in the systematic review for meta-analysis.

### Inclusion and exclusion criteria

The inclusion criteria of the articles for the present systematic review and meta-analysis were original studies involving humans and observational studies such as prospective, case‒control, cohort, and cross-sectional studies. The studies involved participants who were diagnosed with MetS or at risk of the syndrome, including having the risk factors for the syndrome, which are obesity, HTN, hypertriglyceridaemia, low high-density lipoprotein and DM. The studies used human biological samples such as blood and tissues to assess the level of PCBs and their congeners dioxin and furans. The outcome of the studies presented with either MetS or its risk factors. Studies that were included must contain quantitative assessment of the risk, such as odds ratio (OR), hazard ratio (HR) and relative risk (RR) with 95% confidence intervals (CI), mean and standard deviation (SD), mean difference as well as combination of OR with mean and SD. Studies containing in vitro or in vivo studies, review and meta-analysis articles, studies not containing quantitative assessment, studies not related to PCB and its congeners, dioxin and furans and MetS were excluded from the selection. The articles identified were independently screened based on the inclusion and exclusion criteria by two researchers. The final list of eligible articles was discussed by the two researchers and finalized for the present systematic review and meta-analysis.

### Definition of outcomes

MetS is defined based on the criteria established by the World Health Organization (WHO)^29^, European Group for the Study of Insulin Resistance (EGIR) criteria^30^, National Cholesterol Education Program (NCEP) Adult Treatment Panel III (ATP III) criteria^2^ and International Diabetes Foundation (IDF)^31^. The criteria established by the WHO needed impaired fasting glucose (> 100 mg/dl) to be the main criterion for diagnosis, followed by the presence of any two diseases, including obesity (BMI > 30 kg/m^2^), elevated arterial pressure (> 160/90 mmHg), elevated plasma triglycerides (> 1.7 mmol/l), low HDL (< 0.9 mmol/l) and microalbuminuria (albumin:creatinine ratio > 20 mg/g). The EGIR criteria were a modification of the WHO criteria, where the presence of insulin resistance (fasting plasma insulin value > 75th percentile) is the main diagnostic criterion and supported by two diseases, including obesity, HTN or dyslipidemia. The ATP III criteria needed three out of five criteria to be fulfilled to establish MetS in a patient. The five criteria are waist circumference above 40 inches for men and 35 inches for women, blood pressure above 130/85 mmHg, fasting triglyceride level above 150 mg/dl, fasting HDL < 40 mg/dl for men and 50 mg/dl for women and fasting blood glucose level over 100 mg/dl. Last, the IDF criteria described that obesity is needed as the main diagnosis, and this disease is measured by population-specific cut points with additional requirements of two of the four criteria, including fasting glucose above 100 mg/dl, plasma triglyceride level above 150 mg/dl, HDL level below 40 mg/dl for men and 50 mg/dl for women and blood pressure above 130/80 mmHg. Studies that utilized any of the definitions and criteria established by these groups to diagnose the studied population with MetS were included in the present study.

### Data extraction

All eligible studies were independently extracted from the data in a standardized format. The data extracted were arranged in the following order: authors, article title, DOI number, publication year, compound investigated, outcomes measured, country of study, study design, sample size, biological sample type, period of study, detection method, quantitative measurement for each risk factor for MetS such as BMI for obesity, triglyceride level for hypertriglyceridaemia (HTG), blood pressure for HTN, plasma glucose level of DM and the type of statistical analyses performed by each study. The quality of each eligible study was evaluated using the Newcastle–Ottawa Scale^32^. The study was assessed on a scale ranging from 0 to 9, where ratings of 0 to 3, 4 to 6, and 7 to 9 indicated low, moderate, and high quality, respectively.

### Statistical analysis

Meta-analysis was conducted using a random-effects model on the Comprehensive Meta-Analysis V3 platform^33^ to assess the pooled effect estimates to evaluate the association between PCB and its congeners’ exposure with the risk of developing each risk factor for MetS and MetS and dioxin exposure with the risk of developing MetS. Meta-regression analysis was performed to assess the subgroup heterogeneity between moderators. The moderators included in the present meta-regression analysis were countries and year of studies conducted before or after 2015. Funnel plot and Egger's Regression Test were employed to mitigate publication bias and address asymmetry in the research. Publication bias is shown by an asymmetric funnel plot or a P-value < 0.05 in the Egger's Test. The year 2015 was chosen due to the sudden surge of articles being reported in online article databases, which reflects an increase in awareness of EDC exposure.

## Results

### Study results

Based on the PRISMA guidelines, a total of 153 articles were identified from SCOPUS, Web of Science (WoS) and PubMed by the end 2023. From the 153 articles, 59 duplicates were removed from the preliminary screening, leaving 94 articles for screening. Based on the title and abstract screening, 83 articles were excluded, of which 21 articles reported in vivo studies, 6 reported in vitro studies, 16 review papers and 40 articles did not fulfil the inclusion criteria, and EDCs that were not within our scope of review. After exclusion, a total of 11 articles were evaluated on the association between PCBs and dioxin exposure and the risk of developing MetS, including its risk factors. Articles related to furans and their related chemicals were not found in the search results. A systematic review and meta-analysis were carried out focusing on PCBs and dioxin only.

### Study characteristics

Meta-analysis was performed on the 11 studies included in the present investigations on the risk of MetS and its risk factors consisting of obesity, HTN, HTG, low HDL and DM. Out of the 11 studies, seven studies investigated PCB congener exposure, three studies investigated a combination of dioxin and PCB exposure, and one study investigated the effect of dioxin only on the risk of MetS. In MetS-PCB congener exposure, three studies investigated obesity, five studies on HTN, five studies on HTG and four studies on low HDL. The investigation of dioxin exposure and the risk factors for MetS included two studies on DM, two studies on HTN, one study on HTG and one study on low HDL. All studies were conducted between 1998 and 2021 in Sweden, Norway, Spain, South Korea, China and the USA. The sample sizes of these studies ranged from 50 to 35,583 participants. The study design of the included studies consisted of three prospective studies, three cross-sectional studies, two cohort studies and two case‒control studies. The effect size data assessment in six articles was based on the odds ratio (OR), two articles on hazard ratio (HR) and three articles on mean and standard deviation (SD), mean difference and combination of OR with mean and SD, respectively. In terms of samples, 10 articles used blood samples to assess the level of PCB congeners and dioxin, while one study assessed the levels using adipose tissue. The biological samples were taken mostly at the end of the respective study. The detection methods utilized to evaluate the levels of PCB congeners and dioxin were gas chromatography‒mass spectrometry (GC‒MS/MS) and liquid chromatography‒mass spectrometry (LC‒MS/MS) (Table 1).

**Table 1:** Summary of PCB congeners and dioxin investigated in association with MetS.

References	Country	Chemicals	Study design	Study period	Sample size	Sample type	Detection method	Outcomes measured	Quality score	Statistical analysis
Donat-Vargas, Akesson^34^	Sweden	PCB, Dioxin	Prospective	2000–2013	1511	Blood	GC‒MS/MS	MetS	9	Multivariate linear and logistic regression
Dusanov, Ruzzin^35^	Norway	PCB, Dioxin	Cross-sectional	2005–2010	431	Blood	GC‒MS/MS, LC‒MS/MS	Obesity, DM, HTN, HTG, HDL, MetS	9	Binary logistic regression
Dusanov, Svendsen^36^	Norway	PCB, Dioxin	Cross-sectional	2016–2017	120	Blood	LC‒MS/MS, GC‒MS/MS	DM, HTN, HTG, HDL, MetS	7	Mann‒Whitney test
Ha, Kim^37^	Korea	PCB	Case‒control	2016	100	Blood	GC‒MS/MS	Obesity, HTN, HTG, HDL, MetS	9	Multiple logistic regressions
Lind, Salihovic^38^	Sweden	PCB	Prospective	2007–2017	452	Blood	GC‒MS/MS	HTN, HTG, HDL, MetS	9	Gradient-boosted Classification and Regression Trees
Mustieles, Fernandez^39^	Spain	PCB	Cohort	2004–2014	387	Adipose tissue	GC‒MS/MS	MetS	7	Cox-regression models
Pak, Choi^40^	Sweden	Dioxin	Cross-sectional	2016–2021	742	Blood	CALA Assay	DM, MetS	8	Multivariate logistic regression
Rosenbaum, Weinstock^41^	USA	PCB	Cross-sectional	2005–2007	548	Blood	GC‒MS/MS	HTG, Obesity, MetS	7	Logistic regression models
Wallin, Di Giuseppe^42^	Sweden	PCB	Cohort	1998–2012	35,583	Blood	GC‒MS/MS	DM	9	Cox-regression models
Han, Meng^43^	China	PCB	Case‒control	2016–2017	316	Blood	GC‒MS/MS	DM	6	Logistic regression models
Pavuk, Serio^44^	USA	PCB	Prospective	2007—2014	338	Blood	GC‒MS/MS	HTN	9	Logistic regression models

*PCB* polychlorinated biphenyl, *GC‒MS/MS* gas chromatography‒mass spectrometry, *LC‒MS/MS* liquid chromatography‒mass spectrometry, *MetS* metabolic syndrome, *DM* diabetes mellitus, *HTN* hypertension, *HTG* hypertriglyceridaemia, *HDL* low high-density lipoprotein.

### The association between PCB congener exposure and obesity

**Figure 2:**
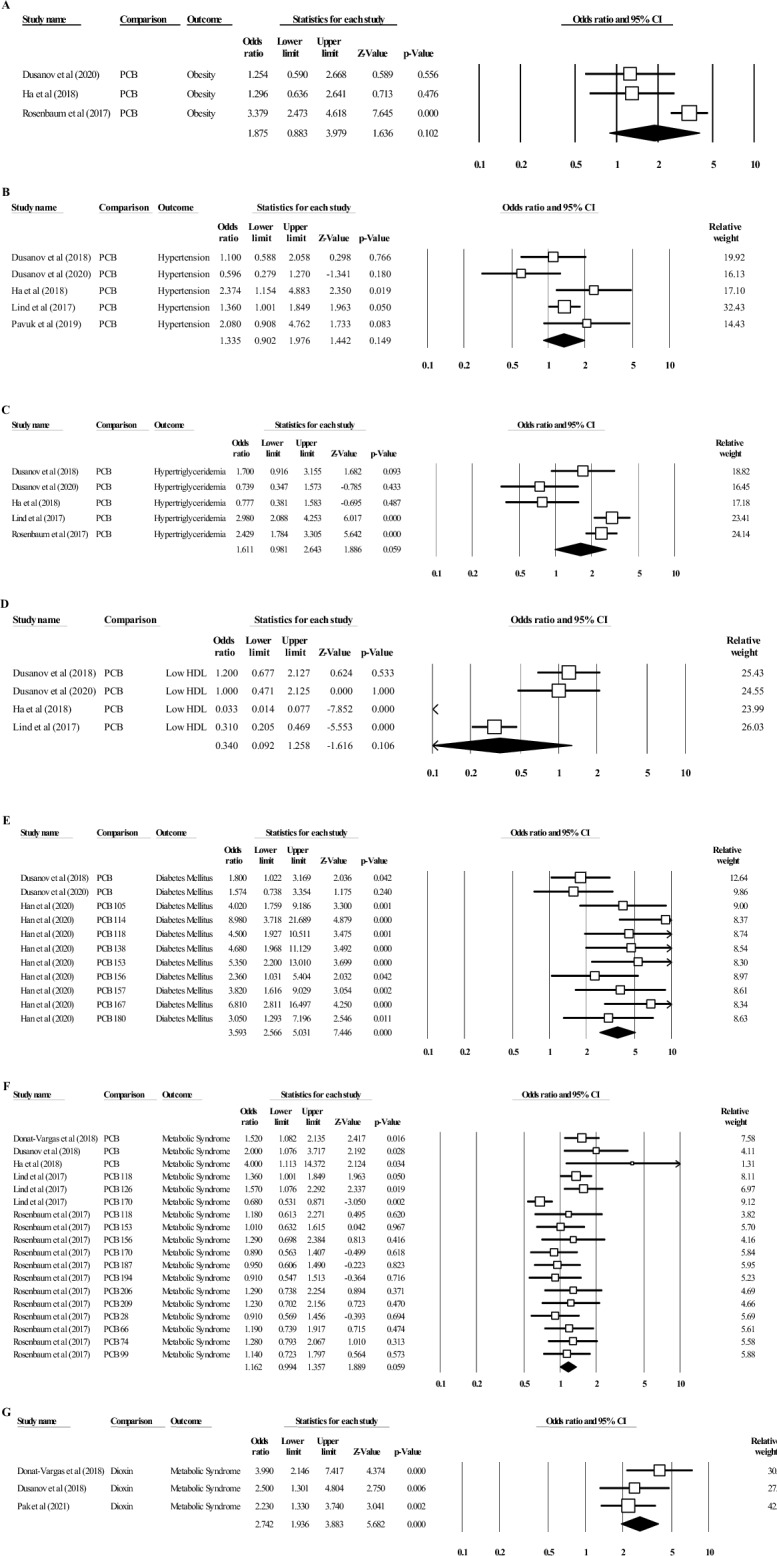
Forest plot of PCB exposure on each risk factor for metabolic syndrome (MetS), including obesity (**A**), hypertension (**B**), hypertriglyceridaemia (**C**), low HDL (**D**), diabetes mellitus (**E**) and MetS (**F**). The forest plot of dioxin exposure and risk of MetS is shown in (**G**). The odds ratio (OR, 95% CI) and relative weight are based on random effects.

Three studies have provided quantitative data to associate the risk of obesity with PCB congener exposure. Two studies reported ORs with 95% CIs, while one study reported the mean difference with a standard deviation of high BMI, indicating overweight and obesity (BMI > 30 kg/m^2^) among the population exposed to PCB in comparison with controls (normal BMI). Based on meta-analysis, the study by Dusanov, Svendsen^36^ showed that PCB congener exposure had a low association with an increase in BMI (OR = 1.254, 95% CI 0.590, 2.668). The study by Ha, Kim^37^ generated an OR of 1.296 (95% CI 0.636, 2.641), and a study by Rosenbaum, Weinstock^41^ produced an OR of 3.379 (95% CI 0.883, 3.979). The study by Rosenbaum, Weinstock^41^ was significant (*P* < 0.05). However, all of these studies have shown a similar association between PCB congener exposure and an increase in BMI. The pooled effects estimated from all three studies showed no statistical significance (*P* = 0.102, random effect) between the association of PCB congener exposure and an increase in BMI. The pooled OR from the meta-analysis provides evidence that the association between the variable and measure outcome has limited evidence of a positive effect (OR = 1.875, 95% CI 0.883, 3.979) of PCB congener exposure causing an increase in BMI, including significantly high heterogeneity (I^2^ = 79.94%, *P* < 0.05) (Table 2, Fig. 2A). Meta-regression based on country and year of study was unable to be executed due to the limited number of studies for the assessment of subgroup heterogeneity.

**Table 2:** Meta-analysis and meta-regression analyses of risk factors for MetS and MetS according to country and year of study from PCB congeners and dioxin exposure.

Risk factors/outcome	Subgroup	Subgroup analysis	Meta-regression	Heterogeneity	
		OR (95% CI)	*P* value	I^2^ (%)	*P* value
Obesity	PCB	1.875 (0.883, 3.979)		79.49	< 0.05
Diabetes mellitus	PCB	3.593 (2.566, 5.031)		46.92	< 0.001
		Region			
	Asia	1.4905 (1.2040, 1.7770)	0.6626	46.92	< 0.05
	Europe	− 0.9509 (− 1.4869, − 0.4148)		0.00	< 0.001
		Year			
	< 2015	0.5878 (− 0.2113, 1.3868)	0.1632	46.92	< 0.05
	> 2015	0.7586 (− 0.0759, 1.6472)		30.71	0.1632
Hypertension	PCB	1.335 (0.902, 1.976)		51.76	< 0.05
		Region			
	Asia	0.8647 (− 0.0394, 7.7687)	0.1893	51.76	0.0608
	Europe	− 0.8009 (− 1.8066, 0.2048)		49.93	0.1186
	Northern America	− 0.1323 (− 1.4742, 1.2096)			0.8468
		Year			
	< 2015	0.3754 (− 0.3989, 1.1497)	0.7861	51.76	0.3420
	> 2015	− 0.1343 (− 1.1043, 0.8357)		63.71	0.7861
Hypertriglyceridaemia	PCB	1.335 (0.902, 1.976)		79.87	< 0.001
		Region			
	Asia	− 0.2524 (− 1.6639, 1.1590)	0.4893	79.87	0.7259
	Europe	0.7484 (− 0.8645, 2.3612)		82.56	0.3631
	Northern America	1.1397 (− 0.7504, 3.0299)			0.2373
		Year			
	< 2015	0.7225 (− 0.2460, 1.6911)	0.4612	79.87	< 0.001
	> 2015	− 0.4778 (− 1.7485, 0.7929)		84.41	< 0.001
Low HDL	PCB	0.340 (0.092, 1.258)		94.48	< 0.001
		Region			
	Asia	− 3.4246 (− 5.1870, − 1.6621)	< 0.001	94.48	< 0.001
	Europe	3.0685 (1.0651, 5.0719)		88.19	< 0.001
		Year			
	< 2015	0.1823 (− 2.5732, 2.9378)	0.2976	94.48	< 0.001
	> 2015	− 1.6952 (− 4.885, 1.4947)		94.37	< 0.001
MetS	PCB	1.162 (0.994, 1.357)		50.63	< 0.05
		Region			
	Asia	1.3863 (0.0321, 2.7405)	0.1330	50.63	< 0.001
	Europe	− 1.1604 (− 2.5388, 0.2181)		50.54	< 0.05
	Northern America	− 1.3066 (− 2.6748, 0.0616)			
		Year			
	< 2015	0.1483 (− 0.0362, 0.3328)	0.9470	50.63	< 0.001
	> 2015	0.0125 (− 0.3555, 0.3804)		51.78	< 0.001
	Dioxin	2.742 (1.936, 3.883)		4.62	< 0.001

### The association between PCB congener exposure and HTN

The association between PCB congener exposure and the risk of HTN was investigated in five studies. Three studies reported ORs^35,38,44^, and two studies^36,37^ reported mean differences with standard deviations. Two studies were found to have a significant P value of less than 0.05 (*P* < 0.05)^37,38^. These studies produced ORs of 2.374 [(95% CI 1.154, 4.883); Ha, Kim^37^] and 1.36 [(95% CI 1.001, 1.849); Lind, Salihovic^38^], suggesting a limited association between exposure to PCB congeners and the risk of HTN. Apart from other studies^35,44^ with ORs above 1 suggesting a low association, a study by Dusanov, Svendsen^36^ suggested that there was an association between PCB exposure and the development of HTN (OR = 0.596, 95% CI 0.279, 1.270). However, the pooled OR showed a lack of association between PCB congener exposure and the risk of HTN (OR = 1.335, 95% CI 0.902, 1.976). The outcome also showed significant medium heterogeneity (I^2^ = 51.76%, *P* < 0.05) (Fig. 2B). Meta-regression analysis showed that subgroup analysis was not significant in either country or year of study (Table 2). Based on country, the heterogeneity was mainly contributed by Asian countries (I^2^ = 51.76%, *P* > 0.05), and studies conducted before 2015 were found to cause heterogeneity (I^2^ = 51.76%, *P* > 0.05).

### The association between PCB congener exposure and HTG

Five studies investigated the association between PCB congener exposure and the risk of HTG. The OR was reported in three studies^35,38,41^, and mean differences with standard deviations were reported in two studies^36,37^. Two studies showed that there was an association between PCB congener exposure and the risk of HTG, with ORs of 0.739 [95% CI 0.347, 1.573; Dusanov, Svendsen^36^] and 0.777 [95% CI 0.381, 1.583; Ha, Kim^37^]. Both of these studies were statistically insignificant (*P* > 0.05). In contrast, three studies showed that there was a low association between PCB congener exposure and outcome^35,38,41^. The pooled OR was 1.611 (95% CI 0.981, 2.643), suggesting no association between PCB congener exposure and the risk of HTG. These studies had significantly high heterogeneity (I^2^ = 79.87%, *P* < 0.001) (Fig. 2C). Heterogeneity based on subgroup analysis showed that the effect was contributed by studies in North America (I^2^ = 82.56%, *P* > 0.05) and studies conducted before 2015 (I^2^ = 79.87%, *P* < 0.001). The heterogeneity for the subgroup was significant for the year of study conducted.

### The association between PCB congener exposure and low HDL level

Four studies presented the association between exposure to PCB congeners and risk of low high-density lipoprotein (HDL) levels. Two studies reported ORs^35,38^, and two studies reported the mean differences with standard deviations^36,37^. Two studies have shown a significant association between the negative effect of PCB congeners and the risk of low HDL (*P* < 0.001). The OR was 0.033 in the study by Ha, Kim^37^ (95% CI 0.014, 0.77) and 0.31 in the study by Lind, Salihovic^38^ (95% CI 0.205, 0.469). The other two studies showed a minimal association between PCB congener exposure and the risk of low HDL. However, the pooled OR (0.34, 95% CI 0.092, 1.258) showed that there was a negative association between PCB congener exposure and the risk of having low HDL. The heterogeneity between the studies was significantly high (I^2^ = 94.48%, *P* < 0.001) (Fig. 2D). Meta-regression analysis showed that European countries contributed to the heterogeneity (I^2^ = 88.19%, *P* < 0.001). Based on the year of study conducted, studies carried out before 2015 contributed to the heterogeneity (I^2^ = 94.48%, *P* < 0.001). Both moderators were found to be significant.

### The association between PCB congener exposure and DM

The association between PCB congener exposure and the risk of DM was investigated in three studies^35,36,43^. Two studies reported the OR between the variable and outcome, while one study reported a mean difference with standard deviation. The study by Han, Meng^43^ reported the OR for each PCB congener, which includes PCBs 105, 114, 118, 138, 153, 156, 157, 167 and 180. Both studies by Dusanov et al.^35,36^ showed a limited association between the risk of DM from PCB congener exposure with ORs of 1.8 (95% CI 1.022, 3.169) and 1.574 (95% CI 0.739, 3.354), respectively. Similarly, the study by Han, Meng^43^ showed no association between DM and PCB congener exposure. The pooled OR showed that PCB congener exposure among the population was highly associated with the risk of DM (OR = 3.593, 95% CI 2.566, 5.031). These studies had significant medium heterogeneity (I^2^ = 46.92%, *P* < 0.001) (Fig. 2E). Asian countries contributed to the heterogeneity effect (I^2^ = 46.92, *P* < 0.05), while studies conducted before 2015 contributed to the effect (I^2^ = 51.76%, *P* > 0.05).

### Association between PCB congener exposure and MetS

Five studies investigated the association between PCB congener exposure and the risk of developing MetS. The diagnosis of MetS was established in the studied population in each study based on fulfilling the criteria established by NCEP ATP III or adjusting for all the risk factors for MetS. Studies by Lind, Salihovic^38^, Rosenbaum, Weinstock^41^ and Ha, Kim^37^ used the NCEP ATP III diagnostic criteria. The study by Donat-Vargas, Akesson^34^ included all risk factors, while the study by Dusanov, Ruzzin^35^ used harmonized criteria to diagnose MetS based on Alberti, Eckel^45^. Based on these diagnostic criteria, four studies reported the OR, and one study reported the mean difference with standard deviation comparing individuals who developed MetS and non-MetS based on PCB congener exposure. From the four studies reporting the OR, two studies stratified the OR based on each PCB congener and risk of MetS. Based on these studies and specific congeners, a study by Lind, Salihovic^38^ showed an association between PCB 170 (OR = 0.68, 95% CI 0.531, 0.871) and the risk of MetS, while a study by Rosenbaum, Weinstock^41^ showed a similar association between PCB 28 (OR = 0.91, 95% CI 0.569, 1.456), PCB 170 (OR = 0.89, 95% CI 0.563, 1.407), PCB 187 (OR = 0.95, 95% CI 0.606, 1.49) and PCB 197 (OR = 0.91, 95% CI 0.547, 1.513). Other studies showed no association between PCB congener exposure and the risk of developing MetS. The pooled OR showed a limited association (OR = 1.162, 95% CI 0.994, 1.357) that was statistically insignificant (*P* > 0.05). All the studies had significant medium heterogeneity (I^2^ = 50.63%, *P* < 0.05) (Fig. 2F). Meta-regression analysis to assess heterogeneity showed that studies carried out in Asian countries contributed to the observed effect (I^2^ = 50.63%, *P* < 0.001). In terms of year of study, studies conducted after 2015 contributed to the heterogeneity (I^2^ = 51.78, *P* < 0.001).

### Association between dioxin exposure and MetS

Three studies investigated the association of dioxin with the risk of developing MetS. The diagnosis of MetS was established using the NCEP ATP III criteria. All studies described the OR. Based on the meta-analysis, the OR from each study showed a minimal association between dioxin exposure and the risk of MetS. The pooled OR described minimal association (OR = 2.742, 95% CI 1.936, 3.883). The heterogeneity was significantly low between studies (I^2^ = 4.62%, *P* < 0.001) (Fig. 2G).

### Assessment of publication bias

**Figure 3:**
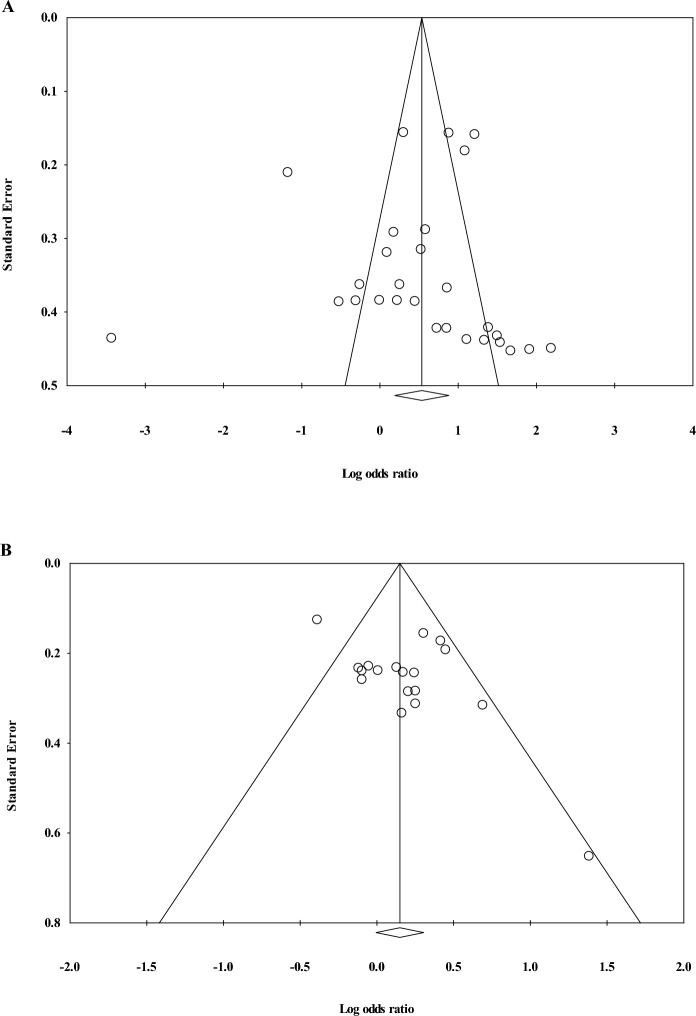
Funnel plot of PCB congener exposure with risk factors for metabolic syndrome (MetS) (**A**) and MetS (**B**).

The included studies were investigated for publication bias based on Egger’s test and funnel plots. Publication bias was assessed based on studies associating MetS risk factors from PCB congener exposure and risk of MetS as the final outcome from PCB congener exposure. The assessment of publication bias based on MetS risk factors from PCB congener exposure showed that the studies included were symmetrically distributed, but 10 variables measured were outside of the funnel plot. Publication bias was not observed with Egger’s test (t = 0.214, *P* = 0.832) (Fig. 3A). The studies associating PCB congener exposure with MetS showed that the studies were within the funnel plot except for one variable. However, based on Egger’s test, publication bias was observed (t = 2.195, *P* = 0.043) (Fig. 3B).

## Discussion

This systematic review and meta-analysis assessed the association between PCB and dioxin exposure and the risk of developing MetS and its risk factors. Eleven studies were included to assess the association. Of the 11 studies, three were prospective studies, four were cross-sectional studies, two were case‒control studies and two were cohort studies.

The mean concentration of PCBs measured in all the studies included was between 7.1 pg/ml and 316 pg/ml. These mean values were reported without correcting to the ratio of total cholesterol and triglyceride values using the empirical equation as described by Bernert, Turner^46^. A study by Mustieles, Fernandez^39^ reported that the mean PCB concentration in lipids was 300 ng/g lipid, while a study by Donat-Vargas, Akesson^34^ reported a mean serum PCB concentration of 42 ng/g lipid, which suggests that the accumulation of PCBs increased with increases in triglyceride and cholesterol levels. A linear trend of plasma lipid levels and accumulation of PCBs has been reported by Aminian, Moinfar^47^, with a strong correlation with PCB 138, 153 and PCB 118. Based on an epidemiological study, the mean concentration of PCBs detected in serum in China was reported to be between 315 ng/g lipid and 8976 ng/g lipid^48^. In contrast, a lower concentration of PCB was reported in another study in China, where the concentration of PCB was reported at 3.5 ng/g lipid with a declining temporal trend after five years of follow-up^49^. In a study conducted in New Jersey, USA, the mean concentration of PCBs was reported to be 65.5 ng/g lipid, and the 95th percentile mean concentration reported was 299 ng/g lipid^50^. Based on these studies, it is suggested that PCB exposure and concentration detected in serum are correlated with the population’s geographical location and the presence of a significant source of PCB exposure in their environmental media. Populations that reside near rivers and use them as sources of drinking water have been shown to have higher concentrations of PCBs in their serum, as PCBs accumulate in the sediments of these riverbeds. The source of PCBs was found from printing factories and construction material markets located along these rivers^51^.

A meta-analysis of PCB exposure and the risk of developing obesity based on the three included studies^36,37,41^ indicated that exposure induced a limited risk of increasing BMI. The congeners of PCBs have been reported to exhibit obesogenic effects on humans. A previous study reported that PCB 170, PCB 180 or PCB 206 exhibited the highest increase in BMI at low to moderate doses and demonstrated inverse effects at higher doses^52^. Similarly, a negative correlation has been observed between PCB 153, PCB 170 and PCB 180 exposure and BMI^53^. The obesogenic effect was observed in in vitro and in vivo studies where exposure to PCBs induced adipocyte hypertrophy through enlargement of lipid droplets and accumulation of PCBs in these droplets^54^. Inhibition of adipocyte differentiation was also observed in these cells with higher concentrations of PCB accumulation^55^. As a result, the increase in BMI results from the expansion and poor differentiation of adipocytes, increasing their body fat percentage.

The risk of HTG and low HDL showed limited association and no association with PCB exposure, respectively. The studies included in the present meta-analysis reported mean HTG levels between 1.1 mmol/l and 1.8 mmol/l. Epidemiological studies have reported that the mean HTG level was approximately 1.7 mmol/l^47,56^, which was consistent with the range of serum HTG levels in the included studies. The increased risk of HTG is most likely due to PCB accumulation in adipocytes leading to expansion of the cells^54^, causing metabolic dysfunction. It has been established that individuals who develop MetS contribute to their unbalanced diet and excessive daily caloric intake^57^. However, the presence and accumulation of PCBs in adipocytes and serum may further increase the risk of HTG by activating NF-κβ signaling, ultimately leading to systemic inflammation and metabolic dysfunction^58^. In addition, leptin signaling in modulating lipid metabolism has been demonstrated to be disrupted with PCB accumulation in adipocytes^59^. These underlying mechanisms have shown that the risk of HTG in MetS patients is most likely contributed by the accumulation of PCB-induced inflammation and metabolic dysfunction, increasing the level of HTG in serum.

For the risk of HTN with PCB exposure, the meta-analysis showed a limited association of being hypertensive upon PCB exposure. The risk of developing HTN was shown to be highly associated with specific congener exposure. A study has demonstrated that PCB 118 and PCB 126 were highly associated with developing risk of HTN compared to PCB 74, PCB 99, PCB 158, 170 and PCB 187^60^. Therefore, in the present meta-analysis, the congeners included in each study involved various congeners, limiting the odds ratio to fully represent the association. However, exposure to PCB congeners, regardless of the congeners, puts an individual at 43% higher risk of developing HTN^61^. The increased risk of HTN from PCB exposure is most likely contributed by PCB-associated atherosclerosis, which has been demonstrated in a few in vivo studies^9,62,63^. Risk factors such as HTG also contribute to the increased risk of HTN.

DM has been shown to have a high association with PCB exposure. The meta-analysis of the included studies showed that the outcome was statistically significant, with an OR above 3.5. The included studies reported mean fasting plasma glucose levels between 5.7 and 8 mmol/l. Based on OR, a study by Han, Meng^43^ showed that PCB 114, PCB 118, PCB 138, PCB 153 and PCB 167 had the highest association with the risk of DM. Several previous studies have reported a positive association between PCB exposure and the risk of DM^64,65^. The underlying mechanisms involving the risk of DM from PCB exposure have been reported to be subsequent outcomes of PCB accumulation leading to adipocyte hypertrophy in the visceral region^66^ and negative alterations of genes regulating insulin secretion from pancreatic cells^67^.

From the studies included in this meta-analysis, five studies reported the OR of MetS as an outcome from PCB exposure. Meta-analysis of these studies has shown limited association between MetS and PCB exposure. The limited association is most likely due to the complexity of establishing MetS in the population, as several diagnostic criteria exist and several risk factors are involved. The complexity of associating PCB exposure with the risk of MetS was found to be contributed by the various congeners, and studies investigating these congeners were often nonuniform and based on their respective objective and population. Despite the limited association, each risk factor assessed in the present meta-analysis showed a certain degree of association except for HDL level. Therefore, it could be ascertained that the risk of MetS begins with PCB exposure, and this occurs through an increase in risk factors. Lee and Shim^68^ hypothesized that the risk of MetS increases with PCB exposure due to atherosclerosis and mitochondrial damage. However, based on the assessment of the OR in the present study, each risk factor exhibited an important role in increasing MetS risk. Risk factors such as DM have a strong association, which suggests an increase in risk initiated by DM followed by other risk factors. The studies included in the present meta-analysis were adjusted for age, BMI, physical activity, education level, cigarette smoking, total energy intake, intake of alcohol, diet type, serum lipid and fasting glucose. Therefore, the OR derived from the present meta-analysis has shown the association and increased risk of MetS from PCB exposure. It is worth mentioning that dioxin was also found to be associated with MetS in the meta-analysis. However, the limited number of studies have not fully proven the association as similar to PCBs, dioxin possesses various isomers and components, making the association a challenge.

The present meta-analysis and meta-regression analysis have shown that PCB exposure is associated with DM patients found in Asian countries. In contrast, HTG was found to be associated with North American countries.

## Strengths and limitations

The relationship between PCBs, dioxins, and MetS holds significant clinical value and potential implications for public health, clinical practice, and policy formulation. By understanding the genetic and environmental interplay associated with PCB and dioxin exposure, it may improve precision medicine it becomes feasible to implement customising clinical management of patients. Individuals with elevated levels of exposure may benefit from more aggressive lifestyle modifications and monitoring. In addition, healthcare professionals should include inquiries about environmental exposure in patients' medical records, particularly for individuals who exhibit MetS or related diseases. This can lead to the development of more comprehensive treatment strategies that consider both lifestyle and environmental factors. One potential approach is to implement enhanced screening programs to identify groups at a greater risk of developing MetS as a result of exposure to PCBs and dioxins. These programs can aid in the timely identification and treatment of MetS. Public health initiatives can be implemented to reduce the risk of exposure to PCBs and dioxins, particularly among communities that are more vulnerable to their harmful effects. This includes regulatory actions aimed at managing and mitigating emissions and contamination of food sources. To reduce the risk of developing MetS among workers in occupations with potential exposure to PCBs and dioxins, it is essential to enforce safety measures and conduct routine health monitoring.

However, due to the limited number of included studies associated with dioxin and MetS, meta-regression analysis was unable to be performed. Among the limitations of conducting the present systematic review and meta-analysis were the limited number of studies investigating MetS patients and their exposure to PCBs. The majority of epidemiological studies were conducted based on each risk factor rather than the outcome (MetS). This posed a limited ability for the authors to select studies, as the independent risk factors may not lead to the development of MetS within the population. In addition, the congeners involved in epidemiological studies were found to have high variance. Thus, high heterogeneity was observed in the included studies in the present meta-analysis. The heterogeneity was due to differences in MetS diagnostic criteria, demographic data of the studied populations, PCB congeners, study period and the risk factors investigated in each study.

## Conclusions

In summary, the present systematic review and meta-analysis was based on 11 studies to assess the association between MetS and its risk factors and PCB and dioxin exposure. Meta-analysis has shown that DM is significantly associated with PCB, whereas MetS and the other risk factors have limited associations with PCB. Dioxin exposure has also been shown to be weakly associated with MetS. The limited number of studies to establish the association between MetS and PCB and dioxin were not adequate for further analysis, and more epidemiological studies investigating MetS as the outcome of disease from PCB and dioxin exposure are needed.

### Supplementary Information

Supplementary Information 1.

Supplementary Information 2.

## Data Availability

The datasets generated and/or analysed during the current study are available from the corresponding author upon reasonable request.
