# Supplementary material for: Association between polychlorinated biphenyl (PCB) and dioxin with metabolic syndrome (METS): a systematic review and meta-analysis
Source: Sci Rep. 2024 Aug 2;14:17941. doi: 10.1038/s41598-024-68369-9 (PMC11297331; doi:10.1038/s41598-024-68369-9)
Supplement: Supplementary file 2 — Supplementary Information 2. [file 41598_2024_68369_MOESM2_ESM.docx]

**SUPPLEMENTARY INFORMATION 2**

**Supplementary Table. Search strategy and the exact words searched in each database**

| Database | Search Within | | | Quick Filters | | | |
| --- | --- | --- | --- | --- | --- | --- | --- |
|  |  |  |  | Publication Years | Document types | Languages | Keyword / Species |
| SCOPUS | ARTICLE TITLE, ABSTRACT, KEYWORDS “persistent organic pollutant” OR dioxin OR “dioxin-like polychlorinated biphenyl” OR “dioxin-like compound” OR “polychlorinated dibenzodioxin” OR “polychlorinated dibenzo-p-dioxin” OR “polychlorodibenzo-4-dioxin” OR PCDD OR TCDD OR tetrachlorodibenzodioxin OR tetrachlorodibenzodioxin OR chlorodibenzofuran OR “polychlorinated dibenzofuran” OR “chlorinated dibenzofuran” OR “polychlorinated biphenyl” OR PCB OR “polychlorobiphenyl compound” | AND | ARTICLE TITLE, ABSTRACT, KEYWORDS “metabolic syndrome” | 2017–2023 | Article | English | Human, humans |
| WoS | TOPIC (persistent organic pollutant) OR (Dioxin OR dioxin-like polychlorinated biphenyl OR dioxin-like compound OR polychlorinated dibenzodioxin OR polychlorinated dibenzo-p-dioxin OR polychlorodibenzo-4-dioxin OR PCDD OR TCDD OR tetrachlorodibenzodioxin OR tetrachlorodibenzodioxin) OR (chlorodibenzofurans OR polychlorinated dibenzofuran OR chlorinated dibenzofuran) OR (polychlorinated biphenyl OR PCB OR polychlorobiphenyl compound) | AND | TOPIC (metabolic syndrome) | 2017–2023 | Article | English | – |
| PubMed | ALL FIELDS ((persistent organic pollutant) OR (Dioxin OR dioxin-like polychlorinated biphenyl OR dioxin-like compound OR polychlorinated dibenzodioxin OR polychlorinated dibenzo-p-dioxin OR polychlorodibenzo-4-dioxin OR PCDD OR TCDD OR tetrachlorodibenzodioxin OR tetrachlorodibenzodioxin) OR (Chlorodibenzofuran OR polychlorinated dibenzofuran OR chlorinated dibenzofuran) OR (polychlorinated biphenyl OR PCB OR polychlorobiphenyl compound)) AND ((metabolic syndrome)) | | | 2017–2023 | Article | English | Human |
